# The local vertex anti-magic coloring for certain graph operations

**DOI:** 10.1016/j.heliyon.2024.e33400

**Published:** 2024-06-27

**Authors:** L. Uma, G. Rajasekaran

**Affiliations:** Department of Mathematics, School of Advanced Sciences, Vellore Institute of Technology, Vellore 632 014, India

**Keywords:** 05C76, 05C78, Local anti-magic coloring, Circulant graphs, Complete bipartite graphs, Null graphs, Join graphs, Complete graphs, Corona product, Tensor product

## Abstract

This work proves the local vertex anti-magic coloring of even regular circulant bipartite graphs C(m;L). Let *G* be either $K_{r,r}$ or $K_{r,r}-F$, *F* is a 1-factor. Also, we discover the local vertex anti-magic coloring for union of bipartite graphs; join graphs G∨H, where $H\in \{O_{r},K_{r},C_{r},K_{r,s}\}$; and the upper bound of corona product $G⊙O_{r}$. It was a problem Lau and Shiu (2023) [1] that: For any $G_{1}$ and $G_{2}$, determine $\chi_{\ell va}(G_{1}\times G_{2})$. We give partial answer to this problem by proving the followings:

1. $\chi_{\ell va}(C_{2m}\times C_{2n})$;
2. $\chi_{\ell va}(C_{2m+1}\times C_{2n+2})$; and
3. $\chi_{\ell va}(P_{3}\times H)$, where $H\in \{K_{r},K_{m,m}\}$.

## 1 Introduction

Throughout the work, the graph (G,V,E) is simple and finite with a vertex set V(G) and an edge set E(G). For an element *a* in V(G), $\omega^{+}(a)=\sum_{e\in E(a)}f(e)$ is the *weight* of *a*, where *f* is an edge labeling of *G*.

Let m∈N and $L\subseteq \{1,2,\ldots ,\lfloor \frac{m}{2}\rfloor \}$. The graph C(m;L) [2] with $V(C(m;L))=Z_{m}$ and $E(C(m;L))=\{i(i+\ell ):i\in Z_{m},\ell \in L\}$ is a *circulant network.* It is evident that $C(m;L)≅K_{m}$ if $L=\{1,2,\ldots ,\lfloor \frac{m}{2}\rfloor \}$. Suppose m>0 is an even integer and $L^{\prime }=\{1,3,5,\ldots ,\frac{m-2}{2}\}$ if m≡0(mod4) or $L^{\prime }=\{1,3,5,\ldots ,\frac{m-4}{2}\}$ if m≡2(mod4). Then, $C(m;\ell_{1},\ell_{2},\ldots ,\ell_{k})$ is the 2*k* -regular circulant bipartite graph if $\ell_{1}$, $\ell_{2}$, … $\ell_{k}\in L^{\prime }$. It is clear that $C(m;L^{\prime })$ is the complete bipartite graph if $L^{\prime }=\{1,3,\ldots ,\frac{m-2}{2}\}$ and m≡0(mod4) or if $L^{\prime }=\{1,3,\ldots ,\frac{m}{2}\}$ and m≡2(mod4). Take note that the number *n* referred as the number of odd lengths in the circulant network. Terminologies and notations not addressed here are accessible in [2], [3].

The *tensor product* $H_{1}\times H_{2}$ [3], of $H_{1}$ and $H_{2}$, is the simple graph with $V(H_{1})\times V(H_{2})$ and the two elements $\left(x_{1},y_{1}\right)$ and $\left(x_{2},y_{2}\right)$ of $H_{1}\times H_{2}$ are adjacent iff $x_{1}∼x_{2}$ in $H_{1}$ and $y_{1}∼y_{2}$ in $H_{2}$. Hence, it is connected ⇔ at least one of the graph is non bipartite.

The *join graph* $G_{1}\vee G_{2}$ [3], of $G_{1}$ and $G_{2}$, is the graph with $V(G_{1}\vee G_{2})=V(G_{1})\cup V(G_{2})$ and $E(G_{1}\vee G_{2})=E(G_{1})\cup E(G_{2})\cup \{ab:a\in V(G_{1})\text{ and }b\in V(G_{2})\}$.

The *corona product* $H_{1}⊙H_{2}$ [3], of $H_{1}$ and $H_{2}$ is the graph obtained by taking one copy of $H_{1}$ and $\left|V(H_{1})\right|$ copies of $H_{2}$ and then join the *i* -th vertex of $H_{1}$ to all the vertices of the *i* -th copy $H_{2}$.

A labeling *ψ* is an *anti-magic coloring* of *G* [4] if there is 1-1 and onto function ψ:E(G)→{1,2,…,|E(G)|} such that $\omega^{+}(a)\neq \omega^{+}(b)$ for all ab∈E(G). It was introduced, in [4], by Hartsfield and Ringel and it was conjectured that: *all connected graph* $G\neq K_{2}$ *is an anti-magic.* Let $P_{m},C_{m},K_{m}$ and $O_{m}$ be, respectively, the path, cycle, complete graph and null graph on *m* vertices. Also, $K_{a,b}$ is the complete bipartite network on a+b elements. For an almost complete survey of graph coloring, see [5].

A function *ψ* is a *local vertex anti-magic coloring* of *G* [6], [7] if an anti-magic coloring *ψ* provides a vertex coloring $\psi^{+}:V(G)\to N$ such that $\psi^{+}(a)\neq \psi^{+}(b)$ for all ab∈E(G). The *local vertex anti-magic chromatic number* $\chi_{\ell va}(G)$ of *G* is min {c(ψ):ψ is local vertex anti-magic chromatic number ofG}. It was introduced, in [7], by Arumugam et al. Also, they posed the following conjectures.

Conjecture 1.1 *[7]**All connected graphs except*$K_{2}$*are local vertex anti-magic.*

Conjecture 1.2 *[7]**Except for*$K_{2}$*, all trees are local vertex anti-magic.*

In [8], Lau et al. found local vertex anti-magic chromatic number as follows:

(i) Assume k≥4, m≥3. If gcd$(n_{s},4k)=1$ and every integer $n_{s}\in (1,2k)$ for every integer s∈[2,m], $\chi_{\ell va}(C(4k;\{1,n_{2},\ldots ,n_{m}\})\vee O_{m})=3$.

(ii) Assume k≥3, gcd(n,4k+2)=1 and an integer n∈(1,2k+1) $\chi_{\ell va}(C(4k+2;\{1,n\})\vee O_{m})=3$.

Also, they proposed the following problems:

**Problem 1.**[8] Suppose $\chi_{\ell va}(H)>\chi (H)$. Determine the circulant network *H* such that (a) $\chi_{\ell va}(H\vee O_{r})=\chi (H)+1$, and (b) $\chi_{\ell va}(H\vee C_{r})=\chi (H)+\chi (C_{r})$.

**Problem 2.**[8] Describe $\chi_{\ell va}((C_{r}\vee C_{s})\vee K_{1})$ for r≠s≥3.

The following Theorems will be used:

Theorem 1.1 *[10]**Let H be a graph with*|V|=p*and*|E|=q*and let*r≥2*and*p≡r(mod2)*. If H has a local vertex anti-magic t-coloring ψ and if either*r≥p*or*p≥2+r*and*$2\psi^{+}(w)\neq p(rp+2q-r^{2}+1)-r(2q+1)$ ∀ w∈V(H)*, then* $\chi_{\ell va}(H\vee O_{r})\leq t+1$*.*

Theorem 1.2 *[13]**Assume that H has a local vertex anti-magic chromatic number it can be colored using colors a and b, where*a<b*. Let A and B represent the number of vertices of colors a and b. Then, H is a bipartite graph whose partitions sizes are A and B with*A>B*and*$Aa=bB=\frac{q(q+1)}{2}$*.*

There have been finite number of studies published by different authors on the local vertex anti-magic chromatic number of join graphs, see [9], [10], [11], [12], [13]. Various writers have published a limited number of results on the local vertex anti-magic chromatic number of corona product see [14], [15], [16], [17]. Let $W_{n}$, n≥3, be a wheel of order *n*. Then, in [18], $\chi_{\ell va}(W_{n}⊙O_{r})=rn+r+3$ if even *n*; and is rn+r+4 if odd *n* (Shankar and Nalliah (2022). Additional information on the local vertex anti-magic chromatic number can be found in [19], [20], [21], [22], [23], [24], [25].

Applications for graph labeling (coloring) can also be in computer science research field like data mining, networking, image recognition, clustering and so on. Creating specific non-periodic codes for pulse radar and missile guidance is similar to labeling the graph with discrete edges. Vertex labels identify the timing pulse transfer.

This study focuses on the local vertex anti-magic chromatic number of even regular bipartite circulant graphs with an arbitrary odd lengths; some join graphs; finite union of bipartite graphs; corona product graphs and tensor product graphs.Additionally, the findings on the graphs of tensor products validate the Problem 4.3 presented in [1]. For integers r<s, [r,s] refers to the set of integers from *r* and *s*. The graph *G* referred as a bipartite throughout this article.

## 2 Results

### 2.1 Bipartite circulant graphs

This section, the local vertex anti-magic chromatic number of bipartite circulant network is presented. In the Theorem 3.3 [26], Lau et al. considered the circulant graph of order 2*m* with odd lengths $1,a_{1},a_{2},\ldots ,a_{t}$, where gcd$(a_{i},2m)=1$, 1≤i≤t. But in our Theorem 2.1, we considered the circulant graph of order 2*m* with arbitrary odd lengths $\ell_{1},\ell_{2},\ldots ,\ell_{k}(\ell_{1}<\ell_{2}<\ldots <\ell_{k})$, and gcd$\left(\ell_{i},2m\right)$ is either 1 or *d*. I.e., gcd$(2m,\ell_{i})=1$ or *d*. Since one of the length is fixed in the Theorem 3.3 [26] but none of the length is fixed in our Theorem 2.1. I.e., $\ell_{1},\ell_{2},\ldots ,\ell_{k}$ are arbitrary odd lengths in the circulant graph. Hence the Theorem 2.1 is more generalization of Theorem 3.3 [26].

Theorem 2.1 *Let* $C(m;L^{\prime \prime })$ *be even regular circulant bipartite graph with* m≥8 *. If* m≡0(mod4) *and* $L^{\prime \prime }\subseteq \left\{1,3,\ldots ,\frac{m-2}{2}\right\}$ *or* m≡2(mod4) *and* $L^{\prime \prime }\subseteq \left\{1,3,\ldots ,\frac{m-4}{2}\right\}$ *, then* $\chi_{\ell va}(C(m;L^{\prime \prime }))=3$ *.*

Proof Let $V(C(m;L^{\prime \prime })=Z_{m}=\{x_{i}:0\leq i\leq m-1\}$ and, for i∈{0,2,…,m−2}, $E(C(m;L^{\prime \prime }))=\{x_{i}x_{i+m-\ell_{j}},x_{i}x_{i+\ell_{j}}:1\leq j\leq n\}$, where subscript taken modulo *m*. Clearly, $|V(C(m;L^{\prime \prime }))|=m$ and $|E(C(m;L^{\prime \prime }))|=mn$. Define $f:E(C(m;L^{\prime \prime }))\to \{1,2,\ldots ,mn\}$ as: For i∈{0,2,…,m−2} and j∈{1,2,…,n},

$$f(x_{i}x_{i+m-\ell_{j}})=\left\{ \begin{array}{cc} \frac{m(2n+1-j)-i}{2} & \text{if}\;\;\text{odd}\;\;\;j,\\ \frac{i+2-m(j-4)}{2} & \text{if}\;\;\text{even}\;\;\;j. \end{array}\right.$$

Next, color the leftover edges of $C(m;L^{\prime \prime })$ as:

$$f(x_{i}x_{i+\ell_{j}})=\left\{ \begin{array}{cc} \frac{i+2+m(j-1)}{2} & \text{if}\;\;\text{odd}\;\;\;j\\ \frac{mj-1}{2} & \text{if}\;\;\text{even}\;\;\;j. \end{array}\right.$$

Thus, *f* is a local vertex anti-magic coloring of $C(m;L^{\prime \prime })$ with vertex colors are: $f^{+}(x_{i})=n(mn+1)$ for i∈{0,2,…,m−2}. For i∈{1,3,…,m−1}, the vertex colors of $x_{i}$ as follows:

If even *n* and $E^{\prime }=\{x_{i}:\ell_{j}\leq i<\ell_{j+1}$, $j\in \{1,3,\ldots ,n-1\}\}\cup \{x_{i}:m-(2+\ell_{j})<i\leq m-(2+\ell_{j-1})$, j∈{2,4,…,n}},

$$f^{+}(x_{i})=\left\{ \begin{array}{cc} \frac{2n(mn+1)-m}{2}+\sum_{j=1}^{n}{\left(-1\right)}^{j}\ell_{j} & \text{for}\;\;\;x_{i}\in E^{\prime },\\ n(mn+1)+\sum_{j=1}^{n}{\left(-1\right)}^{j}\ell_{j} & \text{otherwise.} \end{array}\right.$$

If odd *n*, the vertex color of $x_{i}$ as follows:

Suppose m≡0(mod4), $E^{\prime \prime }=\{x_{i}:\ell_{j}\leq i<\ell_{j+1},j\in \{1,3,\ldots ,n-2\},\ell_{n}\leq i\leq \frac{m-2}{2}\}\cup \{x_{i}:m-(2+\ell_{j})<i\leq m-(2+\ell_{j-1}),j\in \{2,4,\ldots ,n-1\}\}$,

$$f^{+}(x_{i})=\left\{ \begin{array}{cc} n(1+mn)+\sum_{j=1}^{n}{\left(-1\right)}^{j}\ell_{j} & \text{for}\;\;\;x_{i}\in E^{\prime \prime },\\ n(1+mn)+\frac{m}{2}+\sum_{j=1}^{n}{\left(-1\right)}^{j}\ell_{j} & \text{otherwise.} \end{array}\right.$$

Suppose m≡2(mod4), $E^{\prime \prime \prime }=\{x_{i}:\ell_{j}\leq i<\ell_{j+1},j\in \{1,3,\ldots ,n-2\},\ell_{n}\leq i\leq \frac{m}{2}\}\cup \{x_{i}:m-\ell_{j}-2<i\leq m-\ell_{j-1}-2,j\in \{2,4,\ldots ,n-1\}\}$. If $m>1(\ell_{n}+4)$, $E^{0}=\{x_{i}:\frac{m}{2}+2\leq i\leq m-2-\ell_{n}\}$.

$$f^{+}(x_{i})=\left\{ \begin{array}{cc} mn^{2}+n+\sum_{j=1}^{n}{\left(-1\right)}^{j}\ell_{j} & \text{for}\;\;\;x_{i}\in E^{\prime \prime \prime }\cup E^{0},\\ mn^{2}+n+\frac{m}{2}+\sum_{j=1}^{n}{\left(-1\right)}^{j}\ell_{j} & \text{otherwise.} \end{array}\right.$$

**Figure 1:**
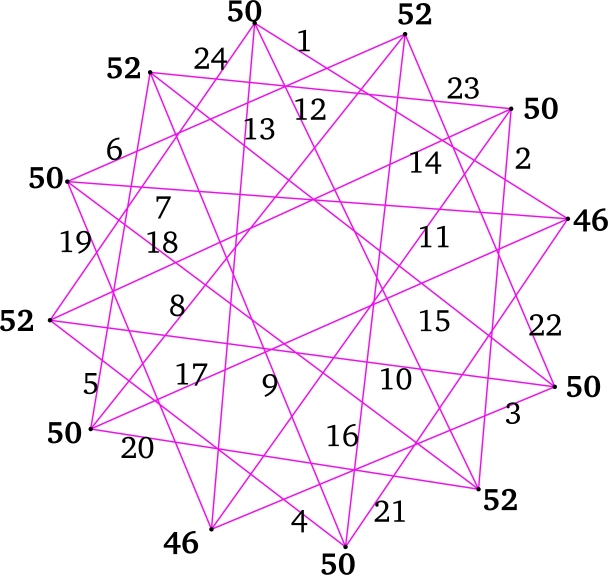
*χ*_*ℓva*_(*C*(12;{3,5}))=3, where gcd(12,3)=3.

Hence $f^{+}$ is an induced vertex coloring of the local vertex anti-magic coloring *f* of $C(m;L^{\prime \prime })$ and $\chi_{\ell va}(C(m;L^{\prime \prime }))\leq 3$. By Theorem 1.2, $\chi_{\ell va}(C(m;L^{\prime \prime }))\geq 3$. Thus, $\chi_{\ell va}(C(m;L^{\prime \prime }))=3$ (refer $\chi_{\ell va}(C(12;\{3,5\}))$ in Fig. 1). □

Corollary 2.1 *[26]* *For every integer* j∈[1,s] *,* $1<b_{1}<\ldots <b_{s}<r$ *and gcd* $(b_{j},2r)=1$ *,* $\chi_{\ell va}(C(2r;\{1,b_{1},\ldots ,b_{s}\}))=3$ *.*

Corollary 2.2 *For* p≥4 *is even,* $\chi_{\ell va}(K_{p,p})=3$ *.*

Hereafter, throughout this article, the graph *G* is even regular bipartite graph with |V|=m. Clearly, *G* is either $K_{\frac{m}{2},\frac{m}{2}}$ if m≡0(mod4) or $K_{\frac{m}{2},\frac{m}{2}}-F$ if m≡2(mod4) and *F* is a 1-factor of length $\frac{m}{2}$. Also, $G≅C(m;L^{\prime })$, where $L^{\prime }=\{1,3,\ldots ,\frac{m-2}{2}\}$ and m≡0(mod4) or $L^{\prime }=\{1,3,\ldots ,\frac{m-4}{2}\}$ and m≡2(mod4).

For r∈N, let $G^{1},G^{2},\ldots ,G^{r}$ be the isomorphic copies of *G* such that $\overset{r}{\underset{k=1}{⋃}}G^{k}=G^{1}\cup G^{2}\cup \ldots \cup G^{r}$. Clearly, $V\left(\overset{r}{\underset{k=1}{⋃}}G^{k}\right)=\{x_{i}^{k}:0\leq i\leq m-1,1\leq k\leq r\}$ and, for i∈{0,2,…,m−2}, $j\in \{1,3,\ldots ,\frac{m-2}{2}\}$, $E\left(\overset{r}{\underset{k=1}{⋃}}G^{k}\right)=\{x_{i}^{k}x_{i+(m-j)}^{k}\}\cup \{x_{i}^{k}x_{i+j}^{k}\}$. Observe that $\left|V\left(\overset{r}{\underset{k=1}{⋃}}G^{k}\right)\right|=rm$ and $\left|E\left(\overset{r}{\underset{k=1}{⋃}}G^{k}\right)\right|=mnr$. Since *n* is the number of all odd lengths of $C(m;L^{\prime })$.

Theorem 2.2 *For*r∈N*,*$\chi_{\ell a}\left(\overset{r}{\underset{k=1}{⋃}}G^{k}\right)=3$*.*

Proof Define $f:E\left(\overset{r}{\underset{k=1}{⋃}}G^{k}\right)\to [1,mnr]$ as follows: For i∈{0,2,…,m−2}, $j\in \{1,3,\ldots ,\frac{m-2}{2}\}$ and k∈{1,2,…,r},

$$f(x_{i}^{k}x_{i+(m-j)}^{k})=\left\{ \begin{array}{cc} \frac{mr(4n+1-j)-2(mk-m+i)}{4} & \;\text{if}\;\;\;j\equiv 1\;(\mathrm{mod}\;4),\\ \frac{mr(4n-1-j)+2(mk-m+2+i)}{4} & \;\text{if}\;\;\;j\equiv 3\;(\mathrm{mod}\;4), \end{array}\right.$$

and

$$f(x_{i}^{k}x_{i+j}^{k})=\left\{ \begin{array}{cc} \frac{mr(j-1)+2m(k-1)+2(i+2)}{4} & \;\text{if}\;\;\;j\equiv 1\;(\mathrm{mod}\;4),\\ \frac{mr(j+1)-2m(k-1)-2i}{4} & \;\text{if}\;\;\;j\equiv 3\;(\mathrm{mod}\;4). \end{array}\right.$$

Hence $f^{+}(x_{i})=n(mnr+1)$ for even *i*.

For *i* is odd, the edge coloring *f* follows by the following cases.

**Case 1.**m≡0(mod4).

$$f^{+}(x_{i})=\left\{ \begin{array}{cc} mn^{2}r & \;\text{if}\;\;\;i\equiv 1\;(\mathrm{mod}\;4),\\ mn^{2}r+2n & \;\text{if}\;\;\;i\equiv 3\;(\mathrm{mod}\;4). \end{array}\right.$$

**Case 2.**m≡2(mod4).

For m≡6(mod8),

$$\begin{array}{ccc} f^{+}(x_{i})=n(mnr+2)+1\; & \;\text{if}\; & \;i\in \{3,7,\ldots ,\frac{m-8}{2}\}\cup \{\frac{m+4}{2},\frac{m+12}{2},\ldots ,m-1\};\\ f^{+}(x_{i})=mn^{2}r\; & \;\text{if}\; & \;i\in \{1,5,\ldots ,\frac{m-4}{2}\}\cup \{\frac{m}{2},\frac{m+8}{2},\ldots ,m-3\}. \end{array}$$

For m≡2(mod8),

$$\begin{array}{ccc} f^{+}(x_{i})=mn^{2}r-1\; & \;\text{if}\; & \;i\in \{1,5,\ldots ,\frac{m-8}{2}\}\cup \{\frac{m+4}{2},\frac{m+12}{2},\ldots ,m-3\};\\ f^{+}(x_{i})=n(mnr+2)+1\; & \;\text{if}\; & \;i\in \{3,7,\ldots ,\frac{m-4}{2}\}\cup \{\frac{m}{2},\frac{m+8}{2},\ldots ,m-1\}. \end{array}$$

**Figure 2:**
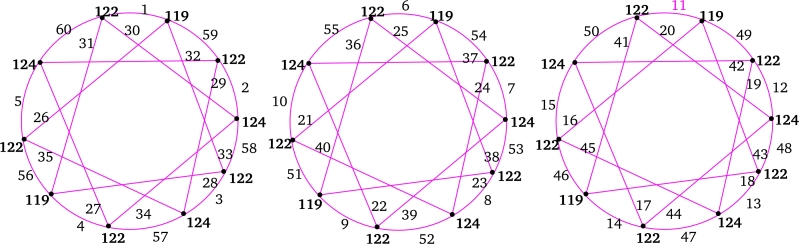
$\chi_{\ell va}(\overset{3}{\underset{k=1}{⋃}}C(10;\{1,3\}))=3$, where *m* = 10, *n* = 2 and *r* = 3.

Therefore, *f* induces a proper vertex coloring $f^{+}$ of $\overset{r}{\underset{k=1}{⋃}}G^{k}$ using 3 colors and $\chi_{\ell a}\left(\overset{r}{\underset{k=1}{⋃}}G^{k}\right)\leq 3$. By Theorem 1.2, $\chi_{\ell va}\left(\overset{r}{\underset{k=1}{⋃}}G^{k}\right)\geq 3$. Hence, $\chi_{\ell va}\left(\overset{r}{\underset{k=1}{⋃}}G^{k}\right)=3$ (refer $\chi_{\ell va}(\overset{3}{\underset{k=1}{⋃}}C(10;\{1,3\}))$ in Fig. 2). □

### 2.2 Join graphs

For circulant networks join with null graphs (isolated vertices), complete, complete bipartite graphs and cycles, we study the local vertex anti-magic coloring in this section.

Let $O_{r}$, r≥1, be the null graph with $V(O_{r})=\{y_{k}:1\leq k\leq r\}$ and let $C(m;\{1,\ell \})\vee O_{r}$ be the join graph with $1<\ell \leq \frac{m-2}{2}$, *m* is even and *ℓ* is odd. Observe that $V(C(m;\{1,\ell \})\vee O_{r})=V(C(m;\{1,\ell \}))\cup V(O_{r})$ and $E(C(m;\{1,\ell \})\vee O_{r})=E(C(m;\{1,\ell \}))\cup \{x_{i}y_{k}:0\leq i\leq m-1,1\leq k\leq r\}$.

In [8], Lau et al. proved that, for m≥12 is even, gcd(ℓ,m)=1 and $1<\ell \leq \frac{m-2}{2}$, $\chi_{\ell va}(C(m;\{1,\ell \})\vee O_{2})=3$. The following Theorem generalizes Theorem 2.1 and 2.6 from [8].

Theorem 2.3 *For*m≥8*,*r≥2*, and gcd*(ℓ,m)=d*,*$\chi_{\ell va}(C(m;\{1,\ell \})\vee O_{r})=3$*.*

Proof Assume that *f* be a local vertex anti-magic coloring of C(m;{1,ℓ}) defined by Theorem 2.1. Then, it provides the vertex coloring *f* as follows:

$$\begin{array}{ccc} f^{+}(x_{i})=2(2m+1)\; & \text{if}\; & i\in \{0,2,\ldots ,m-2\};\\ f^{+}(x_{i})=\frac{7m}{2}+1+\ell \; & \text{if}\; & i\in \{1,3,\ldots ,\ell -2\}\cup \{m-\ell ,m-\ell +2,\ldots ,m-3\};\\ f^{+}(x_{i})=4m++1+\ell \; & \text{if}\; & i\in \{\ell ,\ell +2,\ldots ,m-\ell -2,m-1\}. \end{array}$$

Define an edge function $\varepsilon :E(C(m;1,\ell )\vee O_{r})\to [1,m(r+2)]$ as follows: $\varepsilon (e^{\prime })=f(e^{\prime })$ ∀ $e^{\prime }\in E(C(m;\{1,\ell \}))$. Next, the labeling of the edges $x_{i}y_{k}$ lies between C(m;{1,ℓ}) and $O_{r}$ follows from the cases listed below.

**Case 1.***r* is even.

$$\varepsilon (x_{i}y_{k})=\left\{ \begin{array}{cc} \frac{2m(r+2)-i-mk+m}{2} & \text{if}\;i\in \{0,2,\ldots ,m-2\}\;\text{and}\;k\in \{1,3,\ldots ,r-1\},\\ \frac{2(mr+2m+1)+i-mk}{2} & \text{if}\;i\;\text{and}\;k\;\text{are even.} \end{array}\right.$$

For *i* is odd, the labels of edges $x_{i}y_{1}$ given in the below table: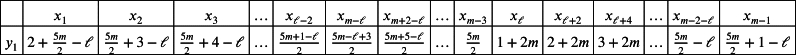 For k∈{3,5,…,r−1},

$$\varepsilon (x_{i}y_{k})=\left\{ \begin{array}{cc} \frac{mk+3m+1+i}{2} & \text{if}\;\;\;i\in \{1,3,\ldots ,\ell -2\},\\ \frac{mk+2m+2\ell +1+i}{2} & \text{if}\;\;\;i\in \{m-\ell ,m-(\ell -2),\ldots ,m-3\},\\ \frac{m(k+3)+\ell +i}{2} & \text{if}\;\;\;i\in \{\ell ,\ell +2,\ldots ,m-(\ell +2)\}. \end{array}\right.$$

For k∈{2,4,…,r},

$$\varepsilon (x_{i}y_{k})=\left\{ \begin{array}{cc} \frac{mk+4m+1-i}{2} & \text{if}\;\;\;i\in \{1,3,\ldots ,\ell -2\},\\ \frac{m(k+5)-2\ell -(i-1)}{2} & \text{if}\;\;\;i\in \{m-\ell ,m-(\ell -2),\ldots ,m-3\},\\ \frac{m(k+4)-\ell -(i-2)}{2} & \text{if}\;\;\;i\in \{\ell ,\ell +2,\ldots ,m-(\ell -+2)\}. \end{array}\right.$$

For 1≤k≤r, the labels of edges $x_{m-1}y_{k}$ given in the below table: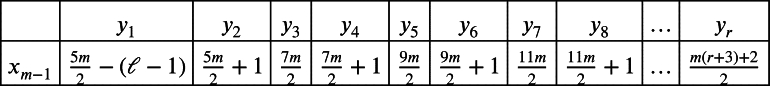 Hence the induced vertex colors of *ε* are as follows:

$$\begin{array}{ccc} \varepsilon^{+}(x_{i})=f^{+}(x_{i})+\frac{mr(3r+8)+2r}{4}\; & \text{if}\; & \;i\in \{0,2,\ldots ,m-2\};\\ \varepsilon^{+}(x_{i})=f^{+}(x_{i})+\frac{m(r^{2}+8r+2)+2(r-2\ell +2)}{4}\; & \text{if}\; & \;i\in \{1,3,\ldots ,\ell -2\}\cup \{m-\ell ,m-(\ell -2),\ldots ,m-3\};\\ \varepsilon^{+}(x_{i})=f^{+}(x_{i})+\frac{mr(r+8)+2(r-2\ell +2)}{4}\; & \text{if}\; & \;i\in \{\ell ,\ell +2,\ldots ,m-(\ell +2),m-1\}. \end{array}$$

$$I.e.,\;\varepsilon^{+}(x_{i})=\left\{ \begin{array}{cc} \frac{8(2m+1)+r(3mr+8m+2)}{4} & \text{if}\;i\;\text{is even},\\ \frac{\left(r+4)(m(r+4)+2\right)}{4} & \text{if}\;i\;\text{is odd}. \end{array}\right.$$

**Case 2.** *r* is odd.

First we assign colors to the edges of the subgraph $C(m;\{1,\ell \})\vee O_{r-2}$ using the labels $\left[2m+1,\frac{m(r+2)}{2}\right]\cup$ $\left[\frac{m(r+6)+2}{2},mr+2m\right]$ by *Case 1.* Next we label the edges $x_{i}y_{r-1}$ and $x_{i}y_{r}$, 0≤i≤m−1, using the labels $\left[\frac{mr+2m+2}{2},\frac{mr+6m}{2}\right]$ as follows:

For i∈{0,2,4,…,m−2}, $\varepsilon (x_{i}y_{r-1})=\frac{m(r+6)-i}{2}$; and $\varepsilon (x_{i}y_{r})=\frac{mr+3m+2i+4}{2}$.

For odd *i*,

$$\varepsilon (x_{i}y_{r-1})=\left\{ \begin{array}{cc} \frac{mr+2m+i+1}{2} & \text{if}\;i\in \{1,3,\ldots ,\ell -2\},\\ \frac{mr+m+1+2\ell +i}{2} & \text{if}\;i\in \{m-\ell ,m-(\ell -2),\ldots ,m-3\},\\ \frac{\ell +mr+2m+i}{2} & \text{if}\;i\in \{\ell ,\ell +2,\ldots ,m-(\ell +2)\}. \end{array}\right.$$

$\varepsilon (x_{m-1}y_{r-1})=\frac{m(r+3)}{2}$ and $\varepsilon (x_{m-1}y_{r})=\varepsilon (x_{m-1}y_{r-1})+1=\frac{m(r+3)+2}{2}$.

Let c=m(r+5). If *i* is odd, the label of edges $x_{i}y_{r}$ are given in the below table: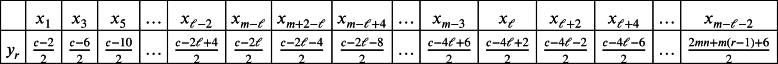 Hence the induced vertex colors of *ε* are as follows:

$$\begin{array}{ccc} \varepsilon^{+}(x_{i})=f^{+}(x_{i})+\frac{mr(3r+8)+2(r+1)-m}{4}\; & \text{if}\; & i\in \{0,2,\ldots ,m-2\};\\ \varepsilon^{+}(x_{i})=f^{+}(x_{i})+\frac{3m+r(8m+mr+2)-2(2\ell -1)}{4}\; & \; & i\in \{1,3,\ldots ,\ell -2\}\cup \{m-\ell ,m-(\ell -2),\ldots ,m-3\};\\ \varepsilon^{+}(x_{i})=f^{+}(x_{i})+\frac{3m+r(8m+mr+2)-4\ell +2}{4}-\frac{m}{2}\; & \text{if}\; & i\in \{\ell ,\ell +2,\ldots ,m-(\ell +2),m-1\}. \end{array}$$

$$\begin{array}{ccc} \text{I.e.},\;\varepsilon^{+}(x_{i})=\frac{8m(r+2)+r(3mr+2)-m+10}{4}\; & \;\text{if}\; & \;i\;\text{is even},\\ \text{I.e.},\;\varepsilon^{+}(x_{i})=\frac{m(r^{2}+8r+17)+2(r+3)}{4}\; & \;\text{if}\; & \;i\;\text{is odd}. \end{array}$$

Hence in both cases, for 1≤k≤r, $\varepsilon^{+}(y_{k})=\frac{m(4m+mr+1)}{2}$.

Therefore, $\varepsilon^{+}$ is an induced vertex coloring of the local vertex anti-magic coloring *ε* of $C(m;\{1,\ell \})\vee O_{r}$ and $\chi_{\ell va}(C(m;\{1,\ell \})\vee O_{r})\leq 3$. Since $\chi_{\ell va}(C(m;\{1,\ell \})\vee O_{r})\geq \chi (C(m;\{1,\ell \})\vee O_{r})=3$. Hence, $\chi_{\ell va}(C(m;\{1,\ell \})\vee O_{r})=3$. □

Corollary 2.3 *[8]**For*m≥3*, gcd*(ℓ,4m)=1*and an integer*ℓ∈(1,2m)*,*$\chi_{\ell va}(C(4m;\{1,\ell \})\vee O_{2})=3$

Corollary 2.4 *[8]**For*m≥3*, gcd*(ℓ,4m+2)=1*and an integer*ℓ∈(1,2m+1)*,*$\chi_{\ell va}(C(4m+2;\{1,\ell \})\vee O_{2})=3$

The following Theorem 2.4, Theorem 2.5, Theorem 2.7 are supports the Problem 4.1 which was proposed in [8] by Lau et al.

Theorem 2.4 *For*r≥2*is even,*$\chi_{\ell va}(G\vee O_{r})=3$*.*

Proof Let *f* be the local vertex anti-magic coloring of *G* defined as in Theorem 2.1. Define an edge mapping $\varepsilon :E(G\vee O_{r})\to [1,m(n+r)]$ as follows: $\varepsilon (e^{\prime })=f(e^{\prime })$ ∀ $e^{\prime }\in E(G)$. Next we label the edges $x_{i}y_{k}$ lies between *G* and $O_{r}$ as follows:

**Case 1.**m≡0(mod4).

Recall that: The induced vertex colors of *f* are:

$$f^{+}(x_{i})=mn^{2}+n\;\text{if even}\;i;f^{+}(x_{i})=mn^{2}-\frac{m}{4}+n\;\text{if}\;i\equiv 1\;(\mathrm{mod}\;4);f^{+}(x_{i})=mn^{2}+n+\frac{m}{4}\;\text{if}\;i\equiv 3\;(\mathrm{mod}\;4).$$

$$\varepsilon (x_{i}y_{1})=\left\{ \begin{array}{cc} \frac{4mn+m+(i+3)}{4} & \text{if}\;\;\;i\equiv 1\;(\mathrm{mod}\;4),\\ \frac{4mn+i+1}{4} & \text{if}\;\;\;i\equiv 3\;(\mathrm{mod}\;4), \end{array}\right.$$

and

$$\varepsilon (x_{i}y_{k})=\left\{ \begin{array}{cc} \frac{2nm+2rm-km+m-i}{2} & \text{if}\;\;\;\text{even}\;\;i\;\;\text{and}\;\;\text{odd}\;\;k,\\ \frac{2mn+2m-km+i+2}{2} & \text{if}\;\;\;\text{even}\;\;i\;\;\text{and}\;\;k,\\ \frac{m(4n+2k)+1-i}{4} & \text{if}\;\;\;i\equiv 1\;(\mathrm{mod}\;4)\;\;\;\text{and}\;\;\text{even}\;\;k,\\ \frac{m(4n+2k-1)+3-i}{4} & \text{if}\;\;\;i\equiv 3\;(\mathrm{mod}\;4)\;\;\;\text{and}\;\;\text{even}\;\;k,\\ \frac{m(4n+2(k-1))+(i+3)}{4} & \text{if}\;\;\;i\equiv 1\;(\mathrm{mod}\;4)\;\;\;\text{and}\;\;\text{odd}\;\;k>1,\\ \frac{m(4n+2k-1)+(1+i)}{4} & \text{if}\;\;\;i\equiv 3\;(\mathrm{mod}\;4)\;\;\;\text{and}\;\;\;\text{odd}\;\;k>1. \end{array}\right.$$

Hence the induced vertex colors of *ε* are as follows:

$$\begin{array}{ccc} \varepsilon^{+}(x_{i})=f^{+}(x_{i})+\frac{rm(4n+3r)+2r}{4}\; & \text{if}\; & i\;\text{is even},\\ \varepsilon^{+}(x_{i})=f^{+}(x_{i})+\frac{mr(4n+r)+m+2r}{4}\; & \text{if}\; & i\equiv 1\;(\mathrm{mod}\;4),\\ \varepsilon^{+}(x_{i})=f^{+}(x_{i})+rmn+\frac{mr(4n+r)-m+2r}{4}\; & \text{if}\; & i\equiv 3\;(\mathrm{mod}\;4). \end{array}$$

**Case 2.** m≡2(mod4).

For m≡6(mod8),

$$\begin{array}{ccc} f^{+}(x_{i})=n(mn+1)\; & \text{if}\; & \text{even}\;i;\\ f^{+}(x_{i})=\frac{4n(mn+1)+m+2}{4}\; & \text{if}\; & i\in \{3,7,\ldots ,\frac{m-8}{2}\}\cup \{\frac{m+4}{2},\frac{m+12}{2},\ldots ,m-1\};\\ f^{+}(x_{i})=\frac{4n(mn+1)-m+2}{4}\; & \text{if}\; & i\in \{1,5,\ldots ,\frac{m-4}{2}\}\cup \{\frac{m}{2},\frac{m+8}{2},\ldots ,m-3\}. \end{array}$$

Next, label the edges $x_{i}y_{k}$ lies between *G* and $O_{r}$ as follows: For $i\in \{\frac{m}{2},\frac{m+8}{2},\ldots ,m-3\}$,

$$\varepsilon (x_{i}y_{k})=\left\{ \begin{array}{cc} \frac{2m(2n+k)-(i+1)}{4} & \text{if}\;\;\;\text{even}\;\;k,\\ \frac{2m(2n+k-1)+i+5}{4} & \text{if}\;\;\;\text{odd}\;\;k>1. \end{array}\right.$$

For $i\in \{\frac{m+4}{2},\frac{m+12}{2},\frac{m+20}{2},\ldots ,m-1\}$,

$$\varepsilon (x_{i}y_{k})=\left\{ \begin{array}{cc} \frac{m(4n+k+1)-i+3}{4} & \text{if}\;\;\;\text{even}\;\;k,\\ \frac{m(4n+2k-1)+i+1}{4} & \text{if}\;\;\;\text{odd}\;\;k>1, \end{array}\right.$$

and

$$\varepsilon (x_{i}y_{1})=\left\{ \begin{array}{cc} \frac{4nm+m+(3+i)}{4} & \text{if}\;\;\;i\in \{\frac{m}{2},\frac{m+8}{2},\ldots ,m-3\},\\ \frac{4nm+m+1+i}{4} & \text{if}\;\;\;i\in \{1,5,\ldots ,\frac{m-4}{2}\},\\ \frac{4mn-1+i}{4} & \text{if}\;\;\;i\in \{\frac{m+4}{2},\frac{m+12}{2},\ldots ,m-1\}. \end{array}\right.$$

Furthermore, the labeling of remaining edges of $G\vee O_{r}$ follows as in the *Case 1.* Hence, the induced vertex colors of *ε* are as follows:

$$\begin{array}{ccc} \varepsilon^{+}(x_{i})=f^{+}(x_{i})+\frac{m(4rn+r^{2}+1)+2(r-1)}{4}\; & \text{if}\; & i\in \{1,5,\ldots ,\frac{m-4}{2}\}\cup \{\frac{m}{2},\frac{m+8}{2},\ldots ,m-3\};\\ \varepsilon^{+}(x_{i})=f^{+}(x_{i})+\frac{m(4rn+r^{2}-1)+2(r-1)}{4}\; & \text{if}\; & i\in \{3,7,\ldots ,\frac{m-8}{2}\}\cup \{\frac{m+4}{2},\frac{m+12}{2},\ldots ,m-1\}. \end{array}$$

For m≡2(mod8), we have the induced vertex colors of $x_{i}$ as follows:

$$\begin{array}{ccc} f^{+}(x_{i})=mn^{2}+n\; & \text{if}\; & \text{even}\;i;\\ f^{+}(x_{i})=\frac{4n(mn+1)+(m-2)}{4}\; & \text{if}\; & i\in \{3,7,\ldots ,\frac{m-4}{2}\}\cup \{\frac{m}{2},\frac{m+8}{2},\ldots ,m-1\};\\ f^{+}(x_{i})=\frac{4n(mn+1)-(m+2)}{4}\; & \text{if}\; & i\in \{1,5,\ldots ,\frac{m-8}{2}\}\cup \{\frac{m+4}{2},\frac{m+12}{2},\ldots ,m-3\}. \end{array}$$

Next, the labeling of the edges $x_{i}y_{k}$ between *G* and $O_{r}$ as follows:

For $i\in \{\frac{m+4}{2},\frac{m+12}{2},\ldots ,m-3\}$,

$$\varepsilon (x_{i}y_{k})=\left\{ \begin{array}{cc} \frac{4mn-(i-1)+2(1+mk)}{4} & \text{if}\;\;\;\text{even}\;\;k,\\ \frac{4mn+2m(k-1)+(1+i)}{4} & \text{if}\;\;\;\text{odd}\;\;k>1. \end{array}\right.$$

For $i\in \{\frac{m}{2},\frac{m+8}{2},\ldots ,m-1\}$,

$$\varepsilon (x_{i}y_{k})=\left\{ \begin{array}{cc} \frac{4mn-(i-3)-m(1-2k)}{4} & \text{if}\;\;\;\text{even}\;\;k,\\ \frac{4mn-m(1-2k)+(1+i)}{4} & \text{if}\;\;\;\text{odd}\;\;k>1, \end{array}\right.$$

and

$$\varepsilon (x_{i}y_{1})=\left\{ \begin{array}{cc} \frac{m(4n+1)+i+3}{4} & \text{if}\;\;\;i\in \{\frac{m+4}{2},\frac{m+12}{2},\ldots ,m-3\},\\ \frac{m(4n+1)+i+5}{4} & \text{if}\;\;\;i\in \{1,5,\ldots ,\frac{m-8}{2}\},\\ \frac{4mn+(i+3)}{4} & \text{if}\;\;\;i\in \{\frac{m}{2},\frac{m+8}{2},\ldots ,m-1\}. \end{array}\right.$$

Furthermore, the labeling of remaining edges of $G\vee O_{r}$ follows as in the *Case 1.* Hence, the induced vertex colors of *ε* are as:

$$\begin{array}{ccc} \varepsilon^{+}(x_{i})=f^{+}(x_{i})+\frac{m(4rn+r^{2}+1)+2(r+1)}{4}\; & \text{if}\; & i\in \{\frac{m+4}{2},\frac{m+12}{2},\ldots ,m-3\},\\ \varepsilon^{+}(x_{i})=f^{+}(x_{i})+\frac{m(4rn+r^{2}-1)+2(r+1)}{4}\; & \text{if}\; & i\in \{\frac{m}{2},\frac{m+8}{2},\ldots ,m-1\}. \end{array}$$

Clearly, in both cases, we have

$$\varepsilon^{+}(x_{i})=\left\{ \begin{array}{cc} \frac{4mn(r+n)+4n+r(3mr+2)}{4} & \text{if}\;i\;\text{is even,}\\ \frac{4mn(r+n)+2(2n+r)+mr^{2}}{4} & \text{if}\;i\;\text{is odd.} \end{array}\right.$$

Moreover, for 1≤k≤r, $\varepsilon^{+}(y_{k})=\frac{m(2mn+mr+1)}{2}$.

**Figure 3:**
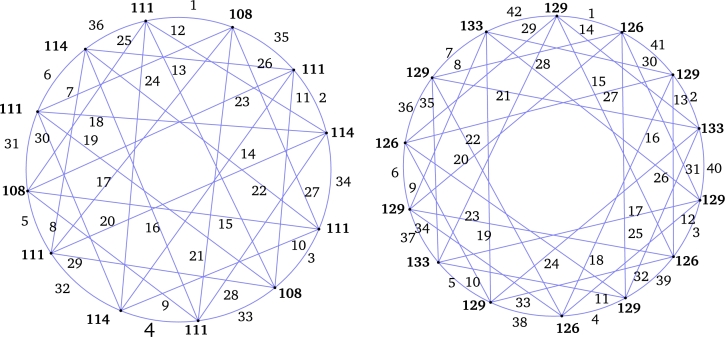
*χ*_*ℓva*_(*C*(12;{1,3,5}))=3 and *χ*_*ℓva*_(*C*(14;{1,3,5}))=3.

Therefore, $\varepsilon^{+}$ is an induced vertex coloring of the local vertex anti-magic coloring *ε* of $G\vee O_{r}$ and $\chi_{\ell va}(G\vee O_{r})\leq 3$. Thus, $\chi_{\ell va}(G\vee O_{r})\geq \chi (G\vee O_{r})=3$. Hence, $\chi_{\ell va}(G\vee O_{r})=3$ (refer the local vertex anti-magic coloring of $C(12;\{1,3,5\})\vee O_{6}$ and $C(14;\{1,3,5\})\vee O_{8}$ is given in Table 1 and Fig. 3. □

**Table 1:**
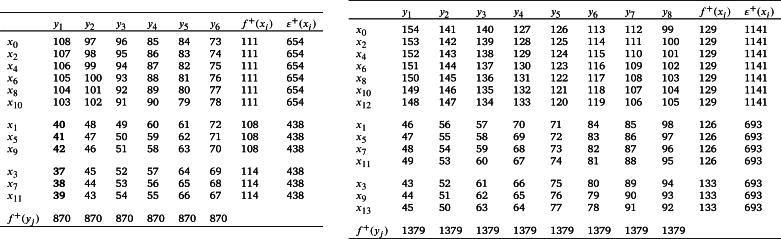
*χ*_*ℓva*_(*C*(12;{1,3,5})∨*O*_6_)=3 and *χ*_*ℓva*_(*C*(14;{1,3,5})∨*O*_8_)=3.

Theorem 2.5 *For*r≥3*is odd,*$\chi_{\ell va}(G\vee O_{r})=3$*.*

Proof First label the edges of the subgraph $G\vee O_{r-2}$ by *ε* using the labels [1,m(n+r−2)] defined by Theorem 2.4. Next label the edges $x_{i}y_{r-1}$ and $x_{i}y_{r}$, 0≤i≤m−1, using the labels [nm+rm−2+1,nm+rm] as follows:

For i∈{0,2,…,m−2}, $\varepsilon (x_{i}y_{r-1})=\frac{2nm+rm+2m-i}{2}$; $\varepsilon (x_{i}y_{r})=\frac{2nm+rm-m+2(i+2)}{2}$.

Now, color the leftover edges of $G\vee O_{r}$ as:

**Case 1.**m≡0(mod4).

$$\varepsilon (x_{i}y_{r-1})=\left\{ \begin{array}{cc} \frac{i+2rm+4m(n-1)+3}{4} & \text{if}\;\;\;i\equiv 1\;(\mathrm{mod}\;4),\\ \frac{m(4n+2r-3)+i+1}{4} & \text{if}\;\;\;i\equiv 3\;(\mathrm{mod}\;4), \end{array}\right.$$

$$\varepsilon (x_{i}y_{r})=\left\{ \begin{array}{cc} \frac{m(2n+r+1)-(i+1)}{2} & \text{if}\;\;\;i\equiv 1\;(\mathrm{mod}\;4),\\ \frac{m(2n+r)-(i-1)}{2} & \text{if}\;\;\;i\equiv 3\;(\mathrm{mod}\;4). \end{array}\right.$$

Hence the induced vertex colors of *ε* are as:

$$\begin{array}{ccc} \varepsilon^{+}(x_{i})=f^{+}(x_{i})+\frac{mr(4n+3r)+2(r+1)-m}{4}\; & \text{if}\; & i\;\text{is even};\\ \varepsilon^{+}(x_{i})=f^{+}(x_{i})+\frac{mr(4n+r)+2(r+m-1)}{4}\; & \text{if}\; & i\equiv 1\;(\mathrm{mod}\;4);\\ \varepsilon^{+}(x_{i})=f^{+}(x_{i})+\frac{mr(r+4n)+2(r-1)}{4}\; & \text{if}\; & i\equiv 3\;(\mathrm{mod}\;4). \end{array}$$

**Case 2.** m≡2(mod4).

For m≡2(mod8),

$$\varepsilon (x_{i}y_{r-1})=\left\{ \begin{array}{cc} \frac{2m(r+2n-2)n+i+3}{4} & \text{if}\;\;\;i\in \{1,5,\ldots ,\frac{m-8}{2}\},\\ \frac{2m(r+2n-2)+i+1}{4} & \text{if}\;\;\;i\in \{\frac{m+12}{2},\frac{m+20}{2},\ldots ,m-3\},\\ \frac{m(4n+2r-3)+i-1}{4} & \text{if}\;\;\;i\in \{3,7,\ldots ,\frac{m-4}{2}\},\\ \frac{m(4n+2r-3)+(1+i)}{4} & \text{if}\;\;\;i\in \{\frac{m}{2},\frac{m+8}{2},\ldots ,m-1\}, \end{array}\right.$$

and

$$\varepsilon (x_{i}y_{r})=\left\{ \begin{array}{cc} \frac{2mn+mr+m-i-1}{2} & \text{if}\;\;\;i\in \{1,5,\ldots ,\frac{m-8}{2}\},\\ \frac{2mn+mr+m-i+1}{2} & \text{if}\;\;\;i\in \{\frac{m+4}{2},\frac{m+12}{2},\ldots ,m-3\},\\ \frac{m(2n+r)-(i-3)}{2} & \text{if}\;\;\;i\in \{3,7,\ldots ,\frac{m-4}{2}\},\\ \frac{m(2n+r)-(i-1)}{2} & \text{if}\;\;\;i\in \{\frac{m}{2},\frac{m+8}{2},\ldots ,m-1\}. \end{array}\right.$$

Hence the induced vertex colors of *ε* are as follows:

$$\begin{array}{ccc} \varepsilon^{+}(x_{i})=f^{+}(x_{i})+\frac{mr(4n+r)+2(r+m)}{4}\; & \text{if}\; & i\in \{1,5,\ldots ,\frac{m-8}{2}\}\cup \{\frac{m+4}{2},\frac{m+12}{2},\ldots ,m-3\};\\ \varepsilon^{+}(x_{i})=f^{+}(x_{i})+\frac{mr(4n+r)+2r}{4}\; & \text{if}\; & i\in \{3,7,\ldots ,\frac{m-8}{2}\}\cup \{\frac{m}{2},\frac{m+8}{2},\ldots ,m-1\}. \end{array}$$

For m≡6(mod8),

$$\varepsilon (x_{i}y_{r-1})=\left\{ \begin{array}{cc} \frac{2m(2n+r-2)+i+3}{4} & \text{if}\;\;\;i\in \{1,5,\ldots ,\frac{m-4}{2}\},\\ \frac{2m(2n+r-2)+i+5}{4} & \text{if}\;\;\;i\in \{\frac{m}{2},\frac{m+8}{2},\ldots ,m-3\},\\ \frac{m(4n+2r-3)+i+3}{4} & \text{if}\;\;\;i\in \{3,7,\ldots ,\frac{m-8}{2}\},\\ \frac{m(4n+2r-3)+i+1}{4} & \text{if}\;\;\;i\in \{\frac{m+4}{2},\frac{m+12}{2},\ldots ,m-1\}, \end{array}\right.$$

and

$$\varepsilon (x_{i}y_{r})=\left\{ \begin{array}{cc} \frac{rm+2nm+m-(1+i)}{2} & \text{if}\;\;\;i\in \{1,5,\ldots ,\frac{m-8}{2}\},\\ \frac{rm+2nm+m-(3+i)}{2} & \text{if}\;\;\;i\in \{\frac{m}{2},\frac{m+8}{2},\ldots ,m-3\},\\ \frac{2nm+rm-(1+i)}{2} & \text{if}\;\;\;i\in \{3,7,\ldots ,\frac{m-4}{2}\},\\ \frac{2nm+rm-i+1}{2} & \text{if}\;\;\;i\in \{\frac{m+4}{2},\frac{m+12}{2},\ldots ,m-1\}. \end{array}\right.$$

Hence the induced vertex colors of *ε* are as follows:

$$\begin{array}{ccc} \varepsilon^{+}(x_{i})=f^{+}(x_{i})+\frac{mr(4n+r)+2(r+m-2)}{4}\; & \text{if}\; & i\in \{1,5,\ldots ,\frac{m-4}{2}\}\cup \{\frac{m}{2},\frac{m+8}{2},\ldots ,m-3\};\\ \varepsilon^{+}(x_{i})=f^{+}(x_{i})+\frac{mr(4n+r)+2(r-2)}{4}\; & \text{if}\; & i\in \{3,7,\ldots ,\frac{m-8}{2}\}\cup \{\frac{m}{2},\frac{m+8}{2},\ldots ,m-1\}. \end{array}$$

Clearly, in both cases, we conclude that,

$$\varepsilon^{+}(x_{i})=\left\{ \begin{array}{cc} \frac{4n(mn+mr+1)+m(3r^{2}-1)+2+2r}{4} & \text{if}\;\;\;i\in \{0,2,\ldots ,m-2\},\\ \frac{4n(mn+mr+1)+m(r^{2}+1)-2+2r}{4} & \text{if}\;\;\;i\in \{1,3,\ldots ,m-1\}. \end{array}\right.$$

Moreover, for 1≤k≤r, $\varepsilon^{+}(y_{k})=\frac{m(2mn+mr+1)}{2}$.

Therefore, $\varepsilon^{+}$ is an induced vertex coloring of the local vertex anti-magic coloring *ε* of $G\vee O_{r}$ and $\chi_{\ell va}(G\vee O_{r})\leq 3$. Thus, $\chi_{\ell va}(G\vee O_{r})\geq \chi (G\vee O_{r})=3$. Hence, $\chi_{\ell va}(G\vee O_{r})=3$. □

The following Proposition 2.1 is very significant to the proof of Theorem 2.6.

Proposition 2.1 *For*r≥3*,*$\chi_{\ell va}(K_{r})=r$*.*

Proof Let $V(K_{r})=\{y_{j}:1\leq j\leq r\}$ and $E(K_{r})=\{y_{j}y_{j^{\prime }}:1\leq j\leq j^{\prime }\leq r\}$. Clearly, $|V(K_{r})|=r$ and $|E(K_{r})|=\frac{r(r-1)}{2}$. Define $\omega :E(K_{r})\to [1,\frac{r(r-1)}{2}]$ as:

$$\begin{array}{ccc} \omega (y_{j}y_{1})=j-1\; & \text{if}\; & j\neq 1,\\ \omega (y_{j}y_{j^{\prime }})=j-1+\sum_{i=2}^{j^{\prime }}r-i\; & \text{if}\; & j>j^{\prime }\;\text{and}\;j^{\prime }\neq 1. \end{array}$$

Thus, *ω* is a local vertex anti-magic coloring of $K_{r}$ with induced vertex colors are:

$$\omega^{+}(y_{j})=\frac{2j^{3}+6j^{2}(1-r)+2j(3r^{2}-3r-4)+3r(3-r)}{6}\;\text{for}\;1\leq j\leq r.$$

Easily, we see that the vertex colors, $\omega^{+}(y_{1})<\omega^{+}(y_{2})<\ldots <\omega^{+}(y_{j})$ are in increasing order. It gives that, $\chi_{\ell va}(K_{r})\leq r$. Since $\chi_{\ell va}(K_{r})\geq \chi (K_{r})=r$. Hence, $\chi_{\ell va}(K_{r})=r$ (refer $\chi_{\ell va}(K_{5})$ in Example 1). □

Example 1 The local anti-magic coloring matrix of $K_{5}$ as follows:
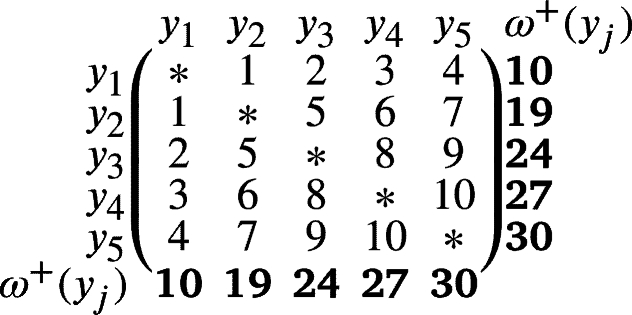

Theorem 2.6 *For*r≥3*,*$\chi_{\ell va}(G\vee K_{r})=r+2$*.*

Proof Let $V(G)=\{x_{i}:0\leq i\leq m-1\}$ and $V(K_{r})=\{y_{k}:1\leq k\leq r\}$.

**Case 1.***r* is even.

Recall that: *ε* is a local vertex anti-magic coloring of $G\vee O_{r}$ (ref. Theorem 2.4), the induced vertex coloring of *ε* is:

$$\varepsilon^{+}(x_{2i})=\frac{4mn(r+n)+4n+r(3mr+2)}{4};\varepsilon^{+}(x_{2i+1})=\frac{4mn(r+n)+2(2n+r)+mr^{2}}{4};\varepsilon^{+}(y_{k})=\frac{m(2mn+mr+1)}{2}.$$

**Case 2.** *r* is odd.

Recall that: *ε* is a local vertex anti-magic coloring of $G\vee O_{r}$(ref. Theorem 2.5), the induced vertex coloring of *ε* is:

$$\varepsilon^{+}(x_{2i})=\frac{4n(mn+mr+1)+m(3r^{2}-1)+2(r+1)}{4},\varepsilon^{+}(x_{2i+1})=\frac{4n(mn+mr+1)+m(r^{2}+1)+2(r-1)}{4},\varepsilon^{+}(y_{k})=\frac{m(2mn+mr+1)}{2}.$$

In both cases, the edge labeling function $\omega :E(K_{r})\to [1,\frac{r(r-1)}{2}]$ is a local vertex anti-magic coloring of $K_{r}$(ref. Proposition 2.1), the induced vertex coloring of *ω* is

$$\omega^{+}(y_{k})=\frac{2k^{3}+6k^{2}(1-r)+2k(3r^{2}-3r-4)+3r(3-r)}{6}.$$

Define $t:E(G\vee K_{r})\to [1,m(r+n)+\frac{r(r-1)}{2}]$ as follows:

$$t(e^{\prime })=\left\{ \begin{array}{cc} \varepsilon (e^{\prime }) & \text{if}\;e^{\prime }\in E(G\vee O_{r});\\ \omega (e^{\prime })+rm+nm & \text{if}\;e^{\prime }\in E(K_{r}). \end{array}\right.$$

Hence we observe that $t^{+}(x_{2i})=\varepsilon^{+}(x_{2i})$, $t^{+}(x_{2i+1})=\varepsilon^{+}(x_{2i+1})$ and

$$t^{+}(y_{k})=\omega^{+}(y_{k})+\varepsilon^{+}(y_{k}),=\frac{2k^{3}+6k^{2}(1-r)+2k(3r^{2}-3r-4)+3r(3-r)}{6}+\frac{m(2mn+mr+1)}{2},=\frac{2k^{3}+6k^{2}(1-r)+2k(3r^{2}-3r-4)+3r(3-r)+3m(2mn+mr+1)}{6}.$$

From the last term $\frac{m(2mn+mr+1)}{2}$ of the above calculation, we have $t^{+}(y_{k})>\varepsilon^{+}(x_{2i})$. Therefore, for 1≤k≤r, $t^{+}(y_{k})>t^{+}(x_{2i})>t^{+}(x_{2i+1})$. Thus, *t* is a local vertex anti-magic coloring of $G\vee K_{r}$ and it induces a vertex coloring $t^{+}$ using r+2 colors. It gives that $\chi_{\ell va}(G\vee K_{r})\leq r+2$. Since $\chi_{\ell va}(G\vee K_{r})\geq \chi (G\vee K_{r})=r+2$. Hence $\chi_{\ell va}(G\vee K_{r})=r+2$. □

Theorem 2.7 *If*s≥3*is odd,*$\chi_{\ell va}(G\vee C_{s})=5$*.*

Proof Clearly, $|V(G\vee C_{s})|=m+s$ and $|E|=|E(G\vee C_{s})|=m(n+s)+s$. Let *ε* be the local vertex anti-magic coloring (ref. Theorem 2.5). Define $\omega :E(G\vee C_{s})\to [1,m(n+s)+s]$ as follows: $\omega (e^{\prime })=\varepsilon (e^{\prime })$ for $e^{\prime }\in E(G\vee O_{s})$, $\omega (y_{k}y_{k+1})=m(n+s)+\frac{k}{2}$ for even *k*, $\omega (y_{k}y_{k+1})=|E|-\frac{\left(k-1\right)}{2}$ for odd *k*. Observe that $\omega^{+}(x_{2i})=\varepsilon^{+}(x_{2i})$ and $\omega^{+}(x_{2i+1})=\varepsilon^{+}(x_{2i+1})$ for $0\leq i\leq \frac{m-2}{2}$. Furthermore, $\omega^{+}(y_{1})=\varepsilon^{+}(y_{1})+\frac{\left(3s+1\right)}{2}$, $\omega^{+}(y_{k})=\varepsilon^{+}(y_{k})+|E|$ for odd k>1 and $\omega^{+}(y_{k})=\varepsilon^{+}(y_{k})+|E|+1$ for even *k*.

For s≥3, *ω* induces 5 distinct vertex colors are as follows:

(i) $\omega^{+}(y_{k})=\frac{m(2mn+ms+1)}{2}+|E|$ if k>1 is odd,

(ii) $\omega^{+}(y_{k})=\frac{m(2mn+ms+1)}{2}+|E|+1$ if *k* is even,

(iii) $\omega^{+}(y_{1})=\frac{m(2mn+ms+1)}{2}+\frac{\left(3s+1\right)}{2}$,

(iv) $\omega^{+}(x_{2i+1})=\frac{4mn(s+n)+2(2n-1)+m+s(ms+2)}{4}$ if $1\leq i\leq \frac{m-2}{2}$ and

(v) $\omega^{+}(x_{2i})=\frac{4mn(s+n)+4(n-1)-m+s(3ms+2)}{4}$ if $0\leq i\leq \frac{m-2}{2}$.

Thus, (i)<(ii)<(iii) and (iv)<(v). Also, (iii)−(iv)$=\omega^{+}(y_{1})-\omega^{+}(x_{2i+1})=$

$$\frac{m(2mn+ms+1)}{2}+\frac{\left(3s+1\right)}{2}-\left[\frac{4mn(s+n)+2(2n-1)+m+s(ms+2)}{4}\right]=\frac{8m^{2}n+2m^{2}s+2m+6s+2-4mns-4mn^{2}-4n+2-m-ms^{2}-2s}{4}=\frac{4mn(2m-n-s)+ms(2m-s)+m+6s+4-4n-2s}{4}=\frac{4mn(2m-n-s)+ms(2m-s)+m-4(n-1)+4s}{4}>0$$

Hence *ω* is a local vertex anti-magic coloring that induces a vertex coloring of $G\vee C_{s}$ using 5 colors, $\chi_{\ell va}(G\vee C_{s})\leq 5$. Therefore, $\chi_{\ell va}(G\vee C_{s})\geq \chi (G\vee C_{s})=5$. □

Theorem 2.8 *Let*m≥8*be a order of G,*r≥2*,*r≡s(mod2)*. For (a)*m+r≤s*(b)*s≤r*,*$\chi_{\ell va}([G\vee O_{r}]\vee O_{s})=\chi_{\ell va}(G\vee K_{r,s})=4$*.*

Proof Proof of this Theorem follows from the Theorem 1.1. □

### 2.3 Corona product of graphs

Consider the corona product $G⊙O_{r}$ with $V(G)\cup \{x_{i}^{k}:0\leq i\leq m-1,1\leq k\leq r\}$ and $E(G)\cup \{x_{i}x_{i}^{k}:0\leq i\leq m-1,1\leq k\leq r\}$.

Theorem 2.9 *Suppose G is of order m and*r≥2*is even,*$\chi_{\ell va}(G⊙O_{r})\leq 2+mr$*.*

Proof Let *f* be a local vertex anti-magic coloring of *G* defined by the Theorem 2.1. Define an edge mapping $\varepsilon :E(G⊙O_{r})\to [1,mn+mr]$ such that $f(e^{\prime })=\varepsilon (e^{\prime })$ for $e^{\prime }\in E(G)$.

Next, label the edges $x_{i}x_{i}^{k}$ lies between *G* and $O_{r}$ as follows: For i∈{0,2,…,m−2},

$$\varepsilon (x_{i}x_{i}^{k})=\left\{ \begin{array}{cc} \frac{2m(r+n)+m(1-k)-i}{2} & \text{if}\;k\in \{1,3,\ldots ,r-1\},\\ \frac{2m(r+n)-mk+2+i}{2} & \text{if}\;k\in \{2,4,\ldots ,r\}. \end{array}\right.$$

Hence the induced vertex colors of *ε* are as follows:

$$\varepsilon^{+}(x_{i})=f^{+}(x_{i})+\frac{r(3mr+4nm+2)}{4}\;\text{for even}\;i,\text{i.e.},\;\varepsilon^{+}(x_{i})=\frac{4mn(n+r)+4n+r(3mr+2)}{4}\;\text{for even}\;i.$$

For i∈{1,3,…,m−1}, k≥3,

$$\varepsilon (x_{i}x_{i}^{k})=\left\{ \begin{array}{cc} \frac{2mn+1+i+m(k-1)}{2} & \text{if}\;\;\;k\in \{3,5,\ldots ,r-1\},\\ \frac{2mn+1-i+mk}{2} & \text{if}\;\;\;k\in \{4,6,\ldots ,r\}. \end{array}\right.$$

Next, label the edges $x_{i}x_{i}^{1}$ and $x_{i}x_{i}^{2}$ lies between *G* and $O_{r}$ follows.

**Case 1.**m≡0(mod4).

$$\varepsilon (x_{i}x_{i}^{1})=\left\{ \begin{array}{cc} \frac{m(4n+1)+3+i}{4} & \text{if}\;\;\;i\in \{1,5,\ldots ,m-3\},\\ \frac{m(4n+1)+3-i}{4} & \text{if}\;\;\;i\in \{3,7,\ldots ,m-1\}, \end{array}\right.$$

and

$$\varepsilon (x_{i}x_{i}^{2})=\left\{ \begin{array}{cc} \frac{4nm+2m+1+i}{4} & \text{if}\;\;\;i\in \{3,7,\ldots ,m-1\},\\ \frac{4m(n+1)-(i-1)}{4} & \text{if}\;\;\;i\in \{1,5,\ldots ,m-3\}. \end{array}\right.$$

Thus, the induced vertex colors of *ε* are as follows:

$$\begin{array}{ccc} \varepsilon^{+}(x_{i})=f^{+}(x_{i})+\frac{r(4mn+mr+2)+m}{4}\; & \text{if}\; & i\equiv 1\;(\mathrm{mod}\;4);\\ \varepsilon^{+}(x_{i})=f^{+}(x_{i})+\frac{r(4mn+mr+2)-m}{4}\; & \text{if}\;i\equiv 3\;(\mathrm{mod}\;4).\; \end{array}$$

**Case 2.** m≡2(mod4).

For m≡6(mod8),

$$\varepsilon (x_{i}x_{i}^{1})=\left\{ \begin{array}{cc} \frac{4mn+1+i}{4} & \text{if}\;\;\;i\in \{3,7,\ldots ,\frac{m-4}{2}\},\\ \frac{2m(2n+1)-(i+1)}{4} & \text{if}\;\;\;i\in \{\frac{m}{2},\frac{m+8}{2},\ldots ,m-3\},\\ \frac{2m(2n+1)-(i-1)}{4} & \text{if}\;\;\;i\in \{1,5,\ldots ,\frac{m-4}{2}\},\\ \frac{4mn-1+i}{4} & \text{if}\;\;\;i\in \{\frac{m+4}{2},\frac{m+12}{2},\ldots ,m-1\}, \end{array}\right.$$

and

$$\varepsilon (x_{i}x_{i}^{2})=\left\{ \begin{array}{cc} \frac{4nm+3m+1+i}{4} & \text{if}\;\;\;i\in \{1,5,\ldots ,\frac{m-8}{2}\},\\ \frac{m(4n+3)-(i-3)}{4} & \text{if}\;\;\;i\in \{\frac{m+4}{2},\frac{m+12}{2},\ldots ,m-1\},\\ \frac{m(4n+3)-5+i}{4} & \text{if}\;\;\;i\in \{3,7,\ldots ,\frac{m-4}{2}\},\\ \frac{m(4n+3)+i+3}{4} & \text{if}\;\;\;i\in \{\frac{m}{2},\frac{m+8}{2},\ldots ,m-3\}. \end{array}\right.$$

Thus, the induced vertex colors of *ε* are as follows:

$$\begin{array}{ccc} \varepsilon^{+}(x_{i})=f^{+}(x_{i})+\frac{r(4mn+mr+2)+m-2}{4}\; & \text{if}\; & i\in \{1,5,\ldots ,\frac{m-4}{2}\}\cup \{\frac{m}{2},\frac{m+8}{2},\ldots ,m-3\};\\ \varepsilon^{+}(x_{i})=f^{+}(x_{i})+\frac{r(4mn+mr+2)-m-2}{4}\; & \text{if}\; & i\in \{3,7,\ldots ,\frac{m-8}{2}\}\cup \{\frac{m+4}{2},\frac{m+12}{2},\ldots ,m-1\}. \end{array}$$

For m≡2(mod8),

$$\varepsilon (x_{i}x_{i}^{1})=\left\{ \begin{array}{cc} \frac{4mn+2m+1-i}{4} & \text{if}\;\;\;i\in \{1,5,\ldots ,\frac{m-8}{2}\},\\ \frac{4mn+3+i}{4} & \text{if}\;\;\;i\in \{\frac{m}{2},\frac{m+8}{2},\ldots ,m-1\},\\ \frac{4mn+1+i}{4} & \text{if}\;\;\;i\in \{3,7,\ldots ,\frac{m-4}{2}\},\\ \frac{2m(2n+1)-(i-3)}{4} & \text{if}\;\;\;i\in \{\frac{m}{2}+2,\frac{m}{2}+6,\ldots ,m-1\}, \end{array}\right.$$

$$\varepsilon (x_{i}x_{i}^{2})=\left\{ \begin{array}{cc} \frac{m(4n+3)+5+i}{4} & \text{if}\;\;\;i\in \{1,5,\ldots ,\frac{m-8}{2}\},\\ \frac{m(4n+3)-(i-3)}{4} & \text{if}\;\;\;i\in \{\frac{m}{2},\frac{m+8}{2},\ldots ,m-1\},\\ \frac{m(4n+3)+5-i}{4} & \text{if}\;\;\;i\in \{3,7,\ldots ,\frac{m-4}{2}\},\\ \frac{m(4n+3)+i+3}{4} & \text{if}\;\;\;i\in \{\frac{m+4}{2},\frac{m+12}{2},\frac{m+20}{2},\ldots ,m-3\}. \end{array}\right.$$

Thus, the induced vertex colors of *ε* are as follows:

$$\begin{array}{ccc} \varepsilon^{+}(x_{i})=f^{+}(x_{i})+\frac{mr(4n+r)+2(r+1)+m}{4}\; & \text{if}\; & i\in \{1,5,\ldots ,\frac{m-8}{2}\}\cup \{\frac{m+4}{2},\frac{m+12}{2},\ldots ,m-3\};\\ \varepsilon^{+}(x_{i})=f^{+}(x_{i})+\frac{mr(4n+r)+2(r+1)-m}{4}\; & \text{if}\; & i\in \{3,7,\ldots ,\frac{m-4}{2}\}\cup \{\frac{m}{2},\frac{m+8}{2},\ldots ,m-1\}. \end{array}$$

Hence in both cases, $\varepsilon^{+}(x_{i})=\frac{4n(mn+1)+r(4mn+mr+2)}{4}$ for *i* is odd,

Clearly, the color of each pendant vertex is the label of the edge incident with that pendant vertex. That is, $\varepsilon^{+}(x_{i}^{k})=\varepsilon (x_{i}x_{i}^{k})$ for k∈{1,2,…,r}, and i∈{1,2,…,m−1}. Hence, $\chi_{\ell va}(G⊙O_{r})\leq 2+mr$ (refer $\chi_{\ell va}(C(14;\{1,3,5\})⊙O_{6})$ in Example 2). □

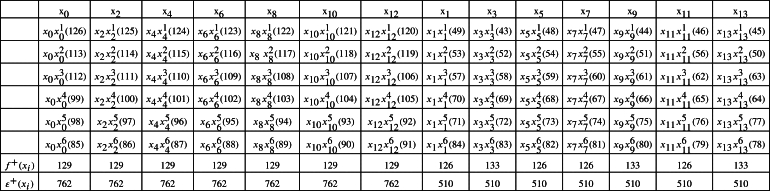

Example 2 $\chi_{\ell va}(C(14;\{1,3,5\})⊙O_{6})\leq 86$

Theorem 2.10 *Suppose G is of order m and*r≥3*is odd,*$\chi_{\ell va}(G⊙O_{r})\leq 2+mr$*.*

Proof First label of edges of the subgraph $G⊙O_{r-2}$ by *ε* (Theorem 2.9) using the labels [1,m(n+r−2)]. Now label the edges $x_{i}\;x_{i}^{r-1}$ and $x_{i}\;x_{i}^{r}$, 0≤i≤m−1, using the labels [nm+rm+1−2m,nm+rm] as follows:

For i∈{0,2,…,m−2}, $\varepsilon (x_{i}\;x_{i}^{r-1})=\frac{2nm+rm+2m-i}{2}$ and $\varepsilon (x_{i}\;x_{i}^{r})=\frac{2nm+rm-m+2(i+2)}{2}$.

For i∈{1,3,…,m−1}, $\varepsilon (x_{i}\;x_{i}^{r-1})=\frac{m(2n+r-2)+i+1}{2}$ and $\varepsilon (x_{i}\;x_{i}^{r})=\frac{m(2n+r+1)-2i}{2}$.

Hence the induced vertex colors of *ε* are as:

$$\varepsilon^{+}(x_{i})=f^{+}(x_{i})+\frac{mr(4n+3r)-m+2(r+1)}{4}\;\text{if}\;i\in \{0,2,\ldots ,m-2\}.\text{I.e.},\;\varepsilon^{+}(x_{i})=mn^{2}+n+\frac{mr(4n+3r)-m+2(r+1)}{4}=\frac{4n(mn+mr+1)+r(3mr+2)-m+2}{4}.$$

Next find the remaining vertex colors of $G⊙O_{r}$ as follows:

For m≡0(mod4),

$$\begin{array}{ccc} \varepsilon^{+}(x_{i})=f^{+}(x_{i})+\frac{mr(4n+r)+2(m-1+r)}{4}\; & \text{if}\; & i\equiv 1\;(\mathrm{mod}\;4)\;\text{and}\\ \varepsilon^{+}(x_{i})=f^{+}(x_{i})+\frac{mr(4n+r)+2(r-1)}{4}\; & \text{if}\; & i\equiv 3\;(\mathrm{mod}\;4). \end{array}$$

For m≡6(mod8),

$$\begin{array}{ccc} \varepsilon^{+}(x_{i})=f^{+}(x_{i})+\frac{mr(4n+r)+2(r+m-2)}{4}\; & \text{if}\; & i\in \{1,5,\ldots ,\frac{m-4}{2}\}\cup \{\frac{m}{2},\frac{m+8}{2},\ldots ,m-2\};\\ \varepsilon^{+}(x_{i})=f^{+}(x_{i})+\frac{mr(4n+r)+2(r-2)}{4}\; & \text{if}\; & i\in \{3,7,\ldots ,\frac{m-8}{2}\}\cup \{\frac{m+4}{2},\frac{m+12}{2},\ldots ,m-1\}; \end{array}$$

For m≡2(mod8),

$$\begin{array}{ccc} \varepsilon^{+}(x_{i})=f^{+}(x_{i})+\frac{mr(4n+r)+2(r+m)}{4}\; & \text{if}\; & i\in \{1,5,\ldots ,\frac{m-8}{2}\}\cup \{\frac{m+4}{2},\frac{m+12}{2},\ldots ,m-3\};\\ \varepsilon^{+}(x_{i})=f^{+}(x_{i})+\frac{mr(4n+r)+2r}{4}\; & \text{if}\; & i\in \{3,7,\ldots ,\frac{m-4}{2}\}\cup \{\frac{m}{2},\frac{m+8}{2},\ldots ,m-1\}. \end{array}$$

Hence, $\varepsilon^{+}(x_{i})=\frac{4n(mn+mr+1)+r(mr+2)+m-2}{4}$ if *i* is odd.

Clearly, the color of each pendant vertex is the label of the edge which incident with that pendant vertex. That is, $\varepsilon^{+}(x_{i}^{k})=\varepsilon (x_{i}x_{i}^{k})$ for k∈{1,2,…,r} and i∈{1,2,…,m−1}.

Thus, $\chi_{\ell va}(G⊙O_{r})\leq 2+mr$. □

### 2.4 Tensor product graphs

This section concentrates of the local vertex anti-magic chromatic number of tensor product graphs. Moreover, the results of this section provides the solution partially to the Problem 4.3 posed in [1].

Theorem 2.11 *For* r≥3 *,* $\chi_{\ell va}(P_{3}\times K_{r})\leq 3$ *.*

Proof Let $V(P_{3}\times K_{r})=\{x_{ij}:1\leq i\leq 3,1\leq j\leq r\}$ and $E(P_{3}\times K_{r})=\{x_{ij}x_{k\ell }:1\leq i<k\leq 3,1\leq j,\ell \leq r,j\neq \ell \}$. Define an edge labeling $\tau :E(P_{3}\times K_{r})\to [1,2r(r-1)]$ as follows:

**Case 1.***r* is odd.

For j<ℓ,

$$\tau (x_{1j}\;x_{2\ell })=\left\{ \begin{array}{cc} 2r^{2}-r\ell -j+1 & \text{if}\;\;j\in \{1,2,\ldots ,r-2\}\;\;\text{and}\;\;\ell \in \{2,4,\ldots ,r-1\},\\ r(2r-\ell -1)+j & \text{if}\;\;j\in \{1,2,\ldots ,r-1\}\;\;\text{and}\;\;\ell \in \{3,5,\ldots ,r\}. \end{array}\right.$$

For j>ℓ,

$$\tau (x_{1j}\;x_{2\ell })=\left\{ \begin{array}{cc} 2r^{2}-r\ell -r-j+1 & \text{if}\;\;j\in \{2,3,\ldots ,r\}\;\;\text{and}\;\;\ell \in \{1,3,\ldots ,r-2\},\\ r(2r-\ell -2)+j & \text{if}\;\;j\in \{3,4,\ldots ,r\}\;\;\text{and}\;\;\ell \in \{2,4,\ldots ,r-1\}. \end{array}\right.$$

For j<ℓ and j∈{1,2,…,r−2},

$$\tau (x_{3j}\;x_{2\ell })=\left\{ \begin{array}{cc} r\ell -2r+j & \text{if}\;\;\ell \in \{2,4,\ldots ,r-1\},\\ r\ell +1-r-j & \text{if}\;\;\ell \in \{3,5,\ldots ,r\}. \end{array}\right.$$

For j>ℓ,

$$\tau (x_{3j}\;x_{2\ell })=\left\{ \begin{array}{cc} r(\ell -1)+j & \text{if}\;\;j\in \{2,3,\ldots ,r\}\;\;\text{and}\;\;\ell \in \{1,3,\ldots ,r-2\},\\ r\ell +1-j & \text{if}\;\;j\in \{3,4,\ldots ,r-1\}\;\;\text{and}\;\;\ell \in \{2,4,\ldots ,r-1\}. \end{array}\right.$$

Hence the induced vertex colors of *τ* are as follows:

For 1≤j≤r,

$$\tau^{+}(x_{1j})=\frac{\left(r-1)(3r^{2}-3r+1\right)}{2};\;\tau^{+}(x_{3j})=\frac{r^{3}-2r^{2}+2r-1}{2}\;\text{and}\;\tau^{+}(x_{2j})=2r^{3}-4r^{2}+3r-1.$$

**Case 2.** *r* is even.

First Label the edges of the subgraph $P_{3}\times K_{r-2}$ using the labels $\left[r^{2}+r+1,2r(r-1)]\cup [1,r(r-3)\right]$ as in the *Case 1*. Next label the edges $e\in E(P_{3}\times K_{r})﹨E(P_{3}\times K_{r-2})$ using the labels [r(r−3)+1,r(r+1)] as follows:

$$\tau (x_{1r}x_{2r-2})=r^{2},\;\tau (x_{3r}x_{2r-2})=r^{2}-2r+1,\;\text{and}$$

$$\tau (e)=\left\{ \begin{array}{cc} r^{2}+1+r-j & e\in \{x_{1j}x_{2r-2}:1\leq j\leq r-2\}\cup \{x_{1j}x_{2r-4}:j\in \{r,r-1\}\},\\ j+r^{2}-3r & e\in \{x_{3j}x_{2r-2}:1\leq j\leq r-2\}\cup \{x_{3j}x_{2r-4}:j\in \{r,r-1\}\}. \end{array}\right.$$

For 1≤j≤r−1, $\tau (x_{1j}x_{2r})=r^{2}+2(j-r)$ and $\tau (x_{3j}x_{2r})=r^{2}-2j+1$.

Hence, the induced vertex colors of *τ* are as follows:

For j∈{1,2,3,…,r},

$$\tau^{+}(x_{1j})=\frac{3r{\left(r-1\right)}^{2}}{2},\;\tau^{+}(x_{3j})=\frac{r^{3}-2r^{2}+3r-2}{2}\;\text{and}\;\tau^{+}(x_{2j})=2r^{3}-4r^{2}+3r-1.$$

**Figure 4:**
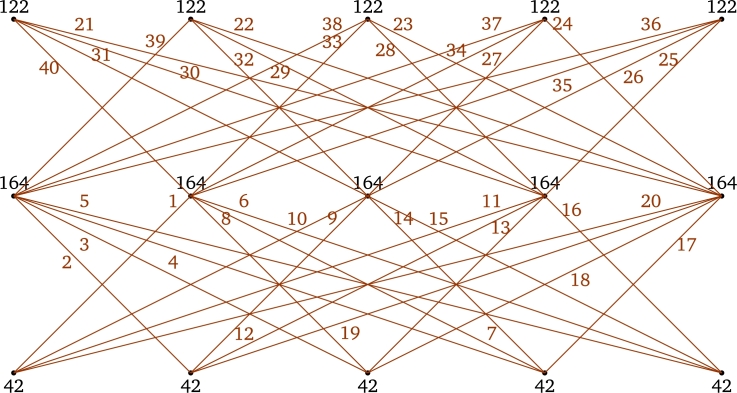
*P*_3_ × *K*_5_ is a bipartite graph and *χ*_*ℓva*_(*P*_3_ × *K*_5_)≤3.

Therefore, $\tau^{+}$ is an induced vertex coloring of the local vertex anti-magic coloring *τ* of $P_{3}\times K_{r}$ and $\chi_{\ell va}(P_{3}\times K_{r})\leq 3$ (refer $\chi_{\ell va}(P_{3}\times K_{5})$ in Fig. 4). □

**Figure 5:**
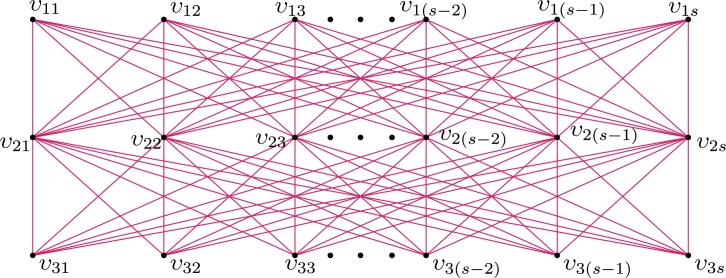
*H* = *K*_*s*,2*s*_.

Let $H≅K_{s,2s}$ (refer *H* in Fig. 5), s≥1 with $|V(K_{s,2s})|=3s$ and $|E(K_{s,2s})|=2s^{2}$. For r∈N, let $H^{1},H^{2},...,H^{r}$ be the isomorphic copies of *H* such that $\overset{r}{\underset{k=1}{⋃}}H^{k}=H^{1}\cup H^{2}\cup \ldots \cup H^{r}$. Clearly, $V\left(\overset{r}{\underset{k=1}{⋃}}H^{k}\right)=\{\upsilon_{ij}^{k}:1\leq i\leq 3,1\leq j\leq s,1\leq k\leq r\}$ and $E\left(\overset{r}{\underset{k=1}{⋃}}H^{k}\right)=\{\upsilon_{1j}^{k}\upsilon_{2j^{\prime }}^{k},\upsilon_{3j}^{k}\upsilon_{2j^{\prime }}^{k}:1\leq j,j^{\prime }\leq s,1\leq k\leq r\}$.

Theorem 2.12 *For*r∈N*,*$\chi_{\ell va}\left(\overset{r}{\underset{k=1}{⋃}}H^{k}\right)\leq 3$*.*

Proof Define $\tau :E\left(\overset{r}{\underset{k=1}{⋃}}H^{k}\right)\to [1,2s^{2}r]$ as follows: For j,ℓ∈{1,2,…,s} and setting a=(k−1)s, we need to deal with two cases.

**Case 1.**s≥2 is even.

$$\tau (\upsilon_{1j}^{k}\upsilon_{2\ell }^{k})=\left\{ \begin{array}{cc} sr(2s-\ell +1)-(j-1)-a & \text{if}\;\;\;\ell \in \{1,3,\ldots ,s-1\},\\ sr(2s-\ell )+j+a & \text{if}\;\;\;\ell \in \{2,4,\ldots ,s\}, \end{array}\right.$$

and

$$\tau (\upsilon_{3j}^{k}\upsilon_{2\ell }^{k})=\left\{ \begin{array}{cc} sr(\ell -1)+j+a & \text{if}\;\;\;\ell \in \{1,3,\ldots ,s-1\},\\ s\ell r-(j-1)-a & \text{if}\;\;\;\ell \in \{2,4,\ldots ,s\}. \end{array}\right.$$

Hence the induced vertex colors of *g* are as follows:

$$\tau^{+}(\upsilon_{1j}^{k})=\frac{s(3s^{2}r+1)}{2},\;\tau^{+}(\upsilon_{3j}^{k})=\frac{s(s^{2}r+1)}{2}\;\text{and}\;\tau^{+}(\upsilon_{2j}^{k})=s(2s^{2}r+1).$$

**Case 2.** s≥3 is odd.

First assign colors to the edges of the subgraph $K_{s-2,s-2}$ using the labels $\left[s^{2}r+2sr+1,2s^{2}r]\cup [1,sr(s-2)\right]$ as in the *Case 1*. Next we label the edges using the labels $\left[(s-2)sr+1,s^{2}r+2sr\right]$ as follows:

$$\tau (\upsilon_{1j}^{k}\upsilon_{2(s-1)}^{k})=sr(s+2)-(j-1)-a;\tau (\upsilon_{1j}^{k}\upsilon_{2s}^{k})=sr(s-1)+2(j+a);\tau (\upsilon_{3j}^{k}\upsilon_{2(s-1)}^{k})=sr(s-2)+j+a\;\text{and}\;\tau (\upsilon_{3j}^{k}\upsilon_{2s}^{k})=sr(s+1)+1-2(j+a).$$

Hence the induced vertex colors of *g* are as follows:

$$\tau^{+}(\upsilon_{1j}^{k})=\frac{3s^{3}r-sr+s+1}{2},\;\tau^{+}(\upsilon_{3j}^{k})=\frac{s^{3}r+sr+s-1}{2}\;\text{and}\;\tau^{+}(\upsilon_{2j}^{k})=s(2rs^{2}+1).$$

Therefore, $\tau^{+}$ is an induced vertex coloring of *τ* of $\overset{r}{\underset{k=1}{⋃}}H^{k}$ and $\chi_{\ell va}\left(\overset{r}{\underset{k=1}{⋃}}H^{k}\right)\leq 3$. □

By substituting s=m and r=2 in Theorem 2.12, we believe that:

Corollary 2.5 *If*m≥2*,*$\chi_{\ell va}(P_{3}\times K_{m,m})\leq 3$*.*

**Figure 6:**
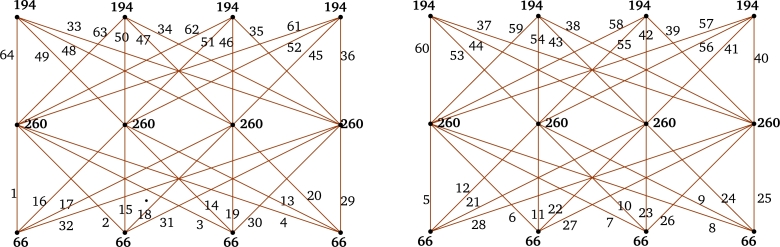
*χ*_*ℓva*_(*P*_3_ × *K*_4,4_)≤3.

The local vertex anti-magic coloring of $P_{3}\times K_{4,4}$ is given in Fig. 6.

Theorem 2.13 *For odd* m≥3 *and even* n≥4 *,* $\chi_{\ell va}(C_{m}\times C_{n})=3$ *.*

Proof Consider $V(C_{m}\times C_{n})=\{(x_{i},y_{j}):1\leq i\leq m,1\leq j\leq n\}$. Define $\tau :E(C_{m}\times C_{n})\to [1,2mn]$ as: For i∈{1,2,…,m} and j∈{1,3,…,n−1},

$$\tau (e)=\left\{ \begin{array}{cc} \frac{2i+mj-m}{2} & \text{if}\;\;\;e=(x_{i},y_{j})(x_{i+1},y_{j+1})\;\;\text{and}\;\;i\neq m,\\ \frac{2i+mj+2(mn-1)-m}{2} & \text{if}\;\;\;e=(x_{i},y_{j})(x_{i+1},y_{j-1}),\;\;i\neq m\;\;\text{and}\;\;j>1,\\ \frac{m(1+4n)-2i+2-mj}{2} & \text{if}\;\;\;e=(x_{i},y_{j})(x_{i-1},y_{j+1})\;\;\text{and}\;\;i>1,\\ \frac{m(1+2n-j)-2i+2}{2} & \text{if}\;\;\;e=(x_{i},y_{j})(x_{i-1},y_{j-1}),\;\;i\neq 1\;\;\text{and}\;\;j>1. \end{array}\right.$$

Furthermore, $\tau ((x_{i},y_{1})(x_{i+1},y_{n}))=mn+i$ if i≠m,

$$\tau ((x_{i},y_{1})(x_{i-1},y_{n}))=mn-i+1\;\text{if}\;i\neq 1,\tau ((x_{1},y_{j})(x_{m},y_{j-1}))=\frac{m(4n+1-j)}{2}\;\text{if}\;j\neq 1,\tau ((x_{1},y_{j})(x_{m},y_{j+1}))=\frac{2mn-(j-1)m}{2},\tau ((x_{1},y_{1})(x_{m},y_{n}))=2mn\;\text{and}\;\tau ((x_{1},y_{n})(x_{m},y_{1}))=m.$$

Hence the induced vertex colors of *τ* are as follows:

$$\tau^{+}(x_{i},y_{j})=\left\{ \begin{array}{cc} 2(2mn+1) & \text{if}\;\;i\in \{1,2,\ldots ,m\}\;\;\text{and}\;\;j\in \{1,3,\ldots ,m-1\},\\ 4mn-2+2m & \text{if}\;\;i\in \{1,m\}\;\;\text{and}\;\;j\in \{2,4,\ldots ,n\},\\ 4mn-2 & \text{otherwise.} \end{array}\right.$$

**Figure 7:**
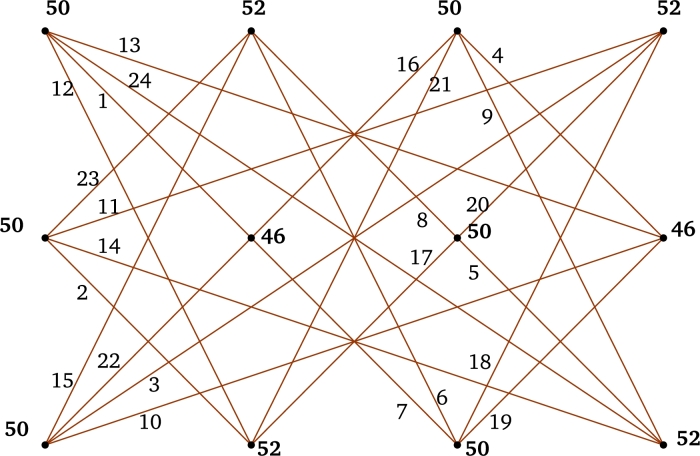
*χ*_*ℓva*_(*C*_3_ × *C*_4_)=3.

Therefore, $\tau^{+}$ is an induced vertex coloring of the local vertex anti-magic coloring *τ* of $C_{m}\times C_{n}$ and $\chi_{\ell va}(C_{m}\times C_{n})\leq 3$. By definition, $C_{m}\times C_{n}$ is a connected bipartite graph with same partite set size, $\chi_{\ell va}(C_{m}\times C_{n})\geq 3$. Hence, $\chi_{\ell va}(C_{m}\times C_{n})=3$ (refer $\chi_{\ell va}(C_{3}\times C_{4})$ in Fig. 7). □

**Figure 8:**
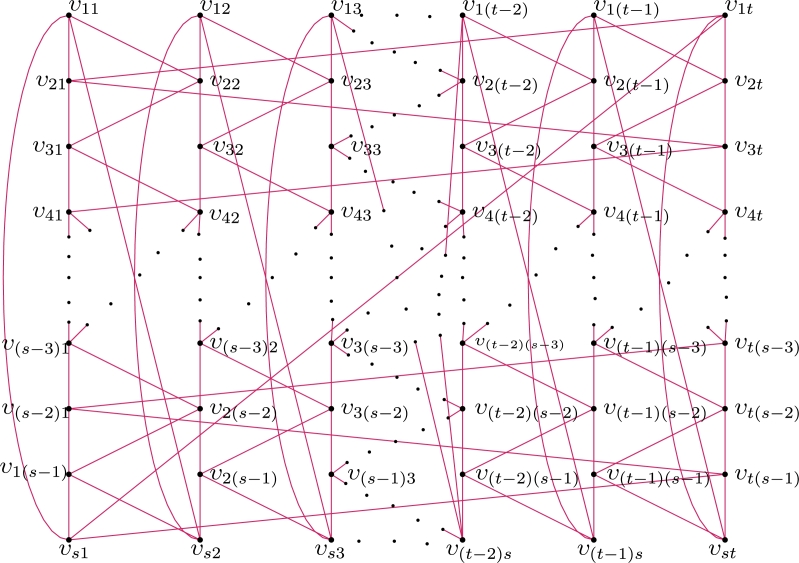
Construction of H.

For r∈N, let $H^{1},H^{2},...,H^{r}$ be isomorphic copies of H (refer H in Fig. 8!) such that $\overset{r}{\underset{k=1}{⋃}}H^{k}=H^{1}\cup H^{2}\cup \ldots \cup H^{r}$. Clearly, s≥4 is even, $V\left(\overset{r}{\underset{k=1}{⋃}}H^{k}\right)=\{\upsilon_{ij}^{k}:1\leq i\leq s,1\leq j\leq t,k\geq 1\}$. Hence, $|V(H^{k})|=rst$ and $|E(H^{k})|=2rst$.

Theorem 2.14 *For*r∈N*,*$\chi_{\ell va}\left(\overset{r}{\underset{k=1}{⋃}}H^{k}\right)=3$*.*

Proof Define $\tau :E\left(\overset{r}{\underset{k=1}{⋃}}H^{k}\right)\to [1,2str]$ as follows: For j∈{1,2,…,t}, k∈{1,2,…,r}, and let $b=\frac{kst-st}{2}$,

$$\tau (\upsilon_{ij}^{k}\upsilon_{(i+1)j}^{k})=\left\{ \begin{array}{cc} \frac{i+sj-s+1}{2}+b & \text{if}\;\;\;\text{odd}\;\;i\;\;\text{and}\;\;1\leq i\leq s-1,\\ \frac{q+s+2-i-sj}{2}-b & \text{if}\;\;\;\text{even}\;\;i\;\;\text{and}\;\;2\leq i\leq s-2, \end{array}\right.$$

$\tau (\upsilon_{1j}^{k}\upsilon_{sj}^{k})=\frac{2q+s+2-i-sj}{2}-b$.

For 1≤j≤t−1,

$$\tau (\upsilon_{ij}^{k}\upsilon_{\left(i+1)(1+j\right)}^{k})=\frac{q+1+i+sj}{2}+b\;\text{if}\;1\leq i\leq s-1\;\tau (\upsilon_{1j}^{k}\upsilon_{s(1+j)}^{k})=\frac{q-sj-s+2}{2}-b.$$

For even *i* and 2≤i≤s−2,

$$\tau (\upsilon_{ij}^{k}\upsilon_{\left(1+i)(j-1\right)}^{k})=\frac{2q-s(j-1)-(i-2)}{2}-b\;\text{if}\;2\leq j\leq t;\tau (\upsilon_{i1}^{k}\upsilon_{(i+1)t}^{k})=\frac{2(q+1)-i}{2}-b;\;\tau (\upsilon_{s1}^{k}\upsilon_{1t}^{k})=\frac{q-s+2}{2}-b.$$

For even *i* and 2≤i≤s,

$$\tau (\upsilon_{i1}^{k}\upsilon_{(i-1)t}^{k})=\frac{q+i}{2}+b.$$

Thus, the induced vertex colors of *τ* are as follows:

For k∈{1,2,…,r} and j∈{1,2,…,t},

$$\tau^{+}(x\upsilon_{ij}^{k})=\left\{ \begin{array}{cc} 2(q+1) & \text{if}\;\;i\in \{2,4,\ldots ,s\},\\ 2(q+2)-s & \text{if}\;\;i=1,\\ 2(q+2) & \text{otherwise.} \end{array}\right.$$

Therefore, *τ* is a local vertex anti-magic coloring and it induces a vertex coloring $\tau^{+}$ with 3 colors. Thus, $\chi_{\ell va}\left(\overset{r}{\underset{k=1}{⋃}}H^{k}\right)\leq 3$. By Theorem 1.2, $\chi_{\ell va}\left(\overset{r}{\underset{k=1}{⋃}}H^{k}\right)\geq 3$. Hence, $\chi_{\ell va}\left(\overset{r}{\underset{k=1}{⋃}}H^{k}\right)=3$. □

By replacing n=2t, s=m and r=2 in Theorem 2.14 we believe that:

Corollary 2.6 *For both*m≥4*and*n≥4*are even,*$\chi_{\ell a}(C_{m}\times C_{n})=3$*.*

**Figure 9:**
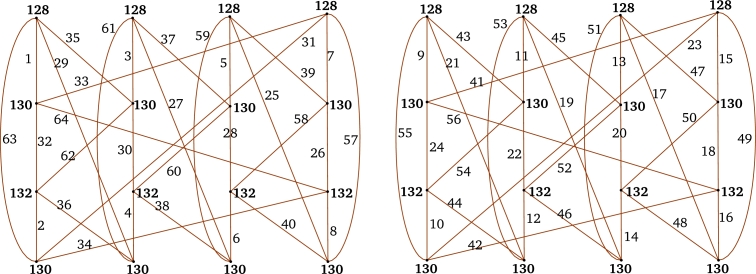
*χ*_*ℓva*_(*C*_4_ × *C*_8_)=3.

The local vertex anti-magic coloring of $C_{4}\times C_{8}$ is given in Fig. 9.

## Conclusion

This work presents the local vertex anti-magic chromatic number of even regular circulant bipartite graphs with arbitrary odd lengths. The local vertex anti-magic chromatic number applies to finite unions of bipartite graphs, join graphs, corona product graphs and tensor product graphs. Finally, we conclude the paper with the challenges listed below.

**Problem 1.** If m≥8, m≡0,2(mod4) and $L^{\prime \prime }\subseteq \{1,3,\ldots ,\frac{m-2}{2}\}$, $\chi_{\ell va}(C(m;L^{\prime \prime })\vee O_{r})=3$.

**Problem 2.** Find $\chi_{\ell va}(P_{n}\times H)$ for any graph *H*.

**Problem 3.** Describe the bipartite graphs *H* for which $\chi_{\ell va}(H)$ is 2 or 3.

Further, we intend to find local vertex anti-magic chromatic number of non-bipartite circulant graphs and product graphs.

## Additional information

No other information used additionally.

## CRediT authorship contribution statement

**L. Uma:** Visualization, Validation, Methodology, Investigation. **G. Rajasekaran:** Writing – original draft, Visualization, Validation, Supervision, Investigation, Conceptualization.

## Declaration of Competing Interest

The authors declare that they have no known competing financial interests or personal relationships that could have appeared to influence the work reported in this paper.

## Data Availability

Not applicable.
